# Supplementary material for: Assessing Gross Motor and Gait Function Using Hip–Knee Cyclograms in Ambulatory Children with Spastic Cerebral Palsy
Source: Sensors (Basel). 2025 Jul 18;25(14):4485. doi: 10.3390/s25144485 (PMC12300134; doi:10.3390/s25144485)
Supplement: Supplementary file 1 [file sensors-25-04485-s001.zip › sensors-3706701-supplementary.pdf]

Supplements

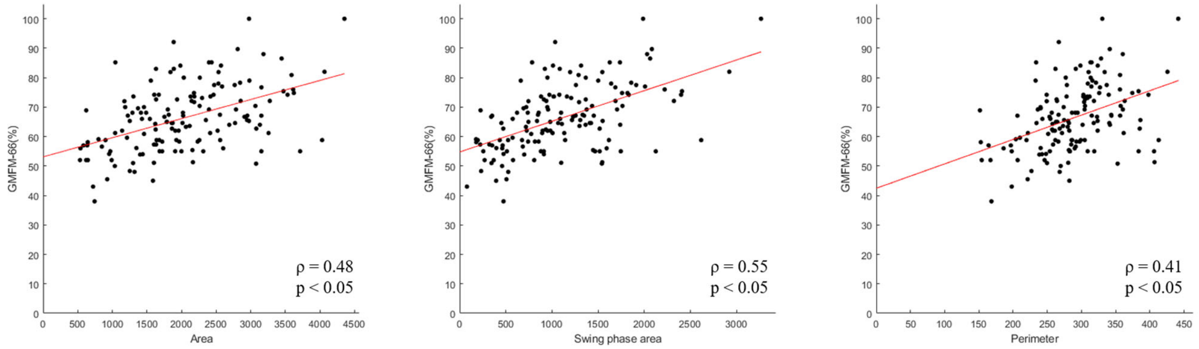

Figure S1. Scatter plot of cyclogram parameters vs GMFM-66.

(GMFM-66: gross motor function measure-66,)

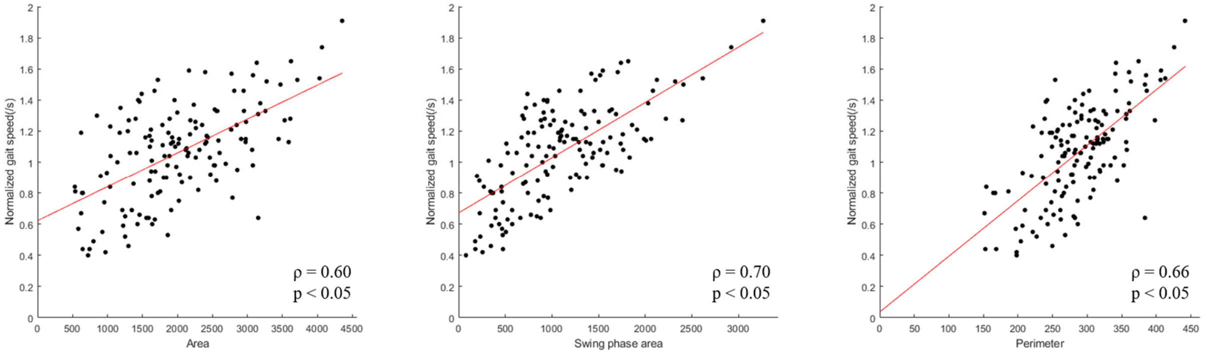

Figure S2. Scatter plot of cyclogram parameters vs normalized gait speed.

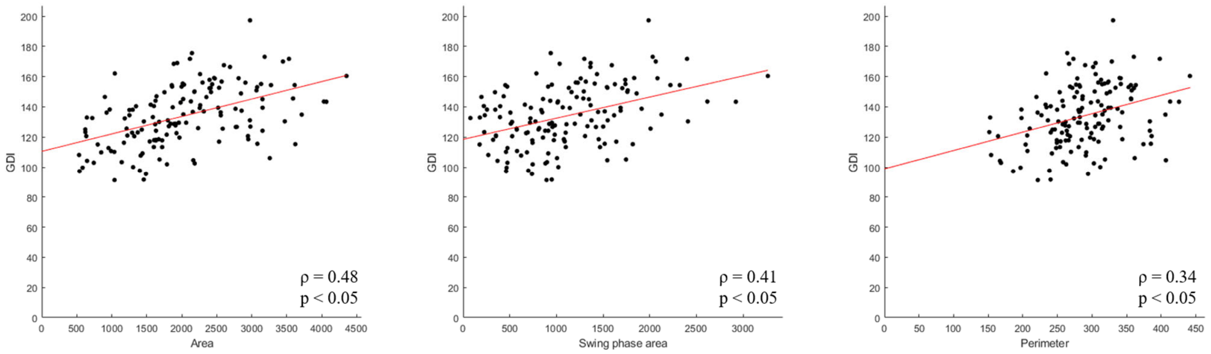

Figure S3. Scatter plot of cyclogram parameters vs GDI ***(GDI: Gait deviation Index).***

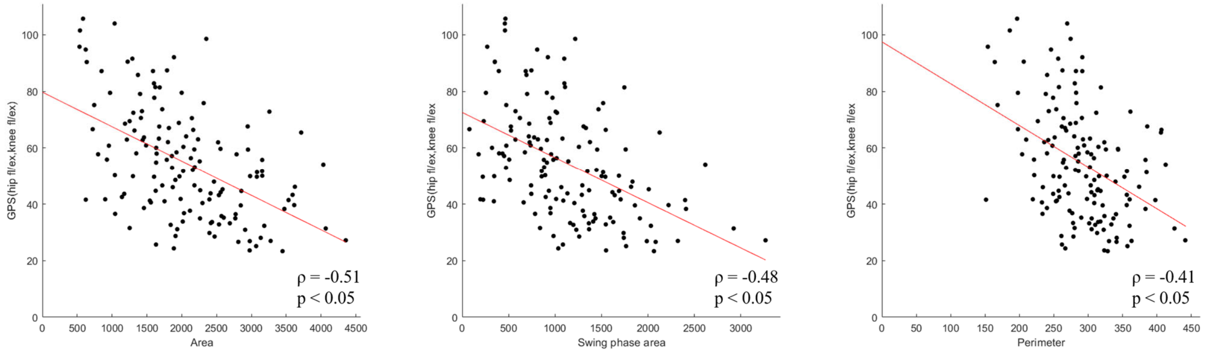

Figure S4. Scatter plot of cyclogram parameters vs GPS(hip fl/ex, knee fl/ex).

(GPS : Gait Profile Score, fl: flexion, ex: extension)

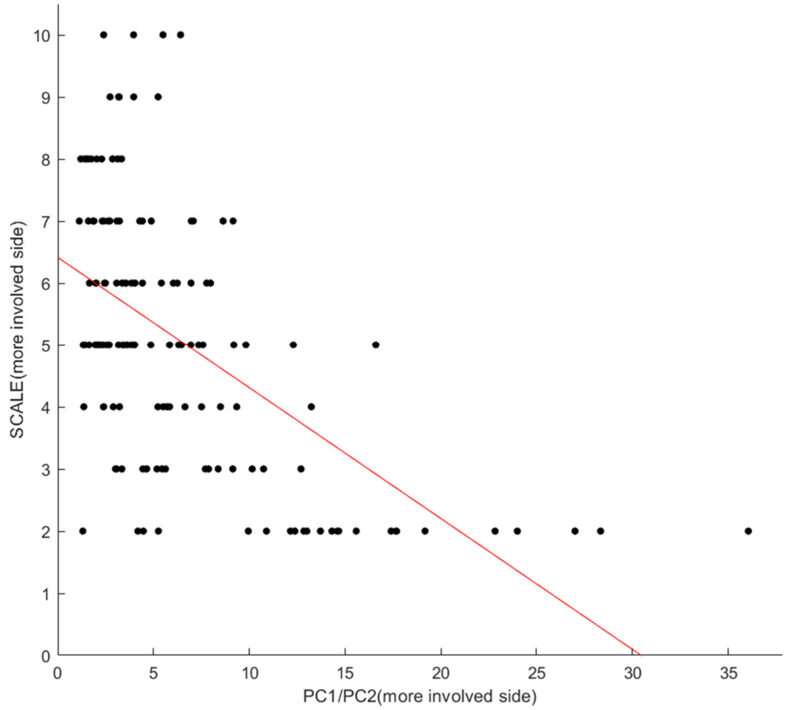

Figure S5. Scatter plot of PC1/PC2 of more invilved side vs SCALE.

(PC : Principle component, SCALE: selective control assessment of the lower extremity)
